# The Effect of Stress on Reproduction and Reproductive Technologies in Beef Cattle—A Review

**DOI:** 10.3390/ani10112096

**Published:** 2020-11-11

**Authors:** Aitor Fernandez-Novo, Sonia S. Pérez-Garnelo, Arantxa Villagrá, Natividad Pérez-Villalobos, Susana Astiz

**Affiliations:** 1 Bovitecnia, Veterinary Consulting, C/Arévalo 5, Colmenar Viejo, 28770 Madrid, Spain; aitorfn@gmail.com; 2 Animal Reproduction Department, Instituto Nacional de Investigación y Tecnología Agraria y Alimentaria (INIA), Avda, Puerta de Hierro s/n, 28040 Madrid, Spain; sgarnelo@inia.es; 3 Centro de Tecnología Animal—Instituto Valenciano de Investigaciones Agrarias (CITA-IVIA), Polígono La Esperanza 100, 12400 Segorbe, Spain; villagra_ara@gva.es; 4 Facultad de Ciencias Biomédicas, Universidad Europea de Madrid, C/Tajo s/n, Villaviciosa de Odón, 28670 Madrid, Spain; natividad.perez@universidadeuropea.es

**Keywords:** stressors, handling stress, thermal stress, reproductive performance, animal welfare

## Abstract

**Simple Summary:**

The economic sustainability of beef herds relies on achieving a reliable percentage of weaned calves. Efforts to lift reproductive efficiency of beef herds have traditionally focused on physical health and nutrition aspects, by extrapolating knowledge from dairy herds. However, animal welfare and stress on beef farms is of outstanding importance. Stress affects the economic sustainability of the farm directly by reducing productive and reproductive performance, as well as indirectly by pushing away customers, who demand farming practices that ensure better animal welfare and lower stress. Despite its relevance and the increasing efforts made by the industry, the detailed ways in which stress influences reproduction of beef herds are still not well understood. Trying to contribute to clarity and reviewing the huge advances made in this topic, we describe the major factors contributing to stress in beef cattle and the effects of that stress on their reproductive performance. We highlight main stressors in beef, such as cattle management, handling stress while passing through the chutes, social hierarchy or weaning effects, besides the nutritional and climate stress and include the acclimatization, acclimation and temperament. We pay attention to the beef bull. And finally, we point out strategies demonstrated to alleviate stressful situations, improving reproductive performance.

**Abstract:**

Researchers have contributed by increasing our understanding of the factors affecting reproduction in beef, mainly physical health and nutrition aspects, which have been main concerns during decades. Animal welfare is of outmost relevance in all animal production systems and it is strongly associated to stress. Stress responses involve endocrine, paracrine and neural systems and the consequences of this stress on the reproductive efficiency of specifically, beef cattle and bulls, need to be highlighted. We, therefore, describe the fundamentals of stress and its quantification, focusing in beef herds, reviewing the highly valuable pieces of research, already implemented in this field. We examine major factors (stressors) contributing to stress in beef cattle and their effects on the animals, their reproductive performance and the success of reproductive biotechnologies. We include terms such as acclimatization, acclimation or temperament, very relevant in beef systems. We examine specifically the management stress due to handling, social environment and hierarchy or weaning effects; nutritional stress; and thermal stress (not only heat stress) and also review the influence of these stressors on reproductive performance and effectiveness of reproductive biotechnologies in beef herds. A final message on the attention that should be devoted to these factors is highlighted.

## 1. Introduction

The economic sustainability of beef herds relies on achieving a reliable percentage of weaned calves. Historically, farmers and veterinarians have worked together to achieve this purpose. Researchers have contributed by increasing our understanding of the factors affecting calving and conception rates in beef herds. Among the main causes of reduced reproductive efficiency in beef, we find infectious diseases [1,2], such as bovine viral diarrhea [3,4], infectious bovine rhinotracheitis [5], trichomoniasis due to *Tritrichomonas foetus* [6,7] and campilobacteriosis due to *Campylobacter foetus spp.* [6]; inadequate nutritional programs [8] which revealed undernutrition and imbalanced nutrition; bull infertility [9], revealing the need to confirm a bull’s breeding soundness before introducing it for natural mating in a beef herd [10]; genetics, with some aspects of reproductive performance in beef cows proving more than 50% heritable [11] and inadequate animal welfare [12]. In contrast to the extensive research on infectious diseases, inadequate nutrition and bull infertility, less is known about how animal welfare interferes with reproductive performance [13,14,15].

Animal welfare refers to the state of an animal as it copes with its environment [16]. An animal’s ability to respond to external stimuli gives it the ability to attain a state of health, prosperity and well-being [17]. Ideally, animals require freedom from thirst, hunger and malnutrition, discomfort, pain, injury, disease, fear and distress; in addition, they should be free to behave normally. Ensuring animal welfare on a beef farm means paying attention to animal social relations, environment, climate and the availability of food and water [14,18]. A loss of welfare stresses animals, which decreases their productive and reproductive herd performance [15,19,20]. At the same time, consumers demand farming practices that ensure better animal welfare and lower stress. Thus, stress affects the economic sustainability of the farm directly by reducing productive and reproductive performance, as well as indirectly by pushing away customers.

Despite its importance, the details of the association between stress and reproductive efficiency of beef herds are still not well understood [15] and need to be highlighted. Stress responses involve endocrine, paracrine and neural systems [21,22] and further work is needed to clarify, in detail, the consequences of the different types of stress on the reproductive abilities of beef cattle and bulls. To take stock of insights so far and to highlight questions for future research, the present review examines the major factors contributing to stress in beef cattle and the effects of that stress on the animals, their reproductive performance and the success of reproductive biotechnologies.

## 2. Definitions, Physiology and Quantification of Stress

### 2.1. Stress Basics

**Figure 1:**
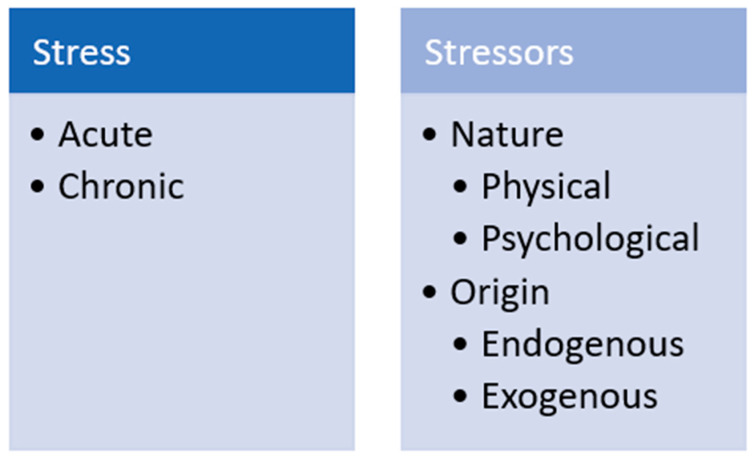
Stress and stressor classification.

Hans Seyle provided perhaps the first definition of “stress” in 1936 [23], calling it a non-specific response of the body to any demand. Since then, the concept has been expanded [24,25], for example, defined it as a biological response elicited when an individual perceives a threat (“stressor”) to its homeostasis. Collier and colleagues proposed perhaps one of the most widely accepted definitions of stress as the result of an external event or condition (stressor) that places a strain on a biological system [24]. Stressors can be physical, such as heat, noise, transportation and deprivation or restriction of food. Stressors can also be psychological, such as weaning, social isolation or mixing, restraint and handling [18]. Stressors can also be classified as endogenous, if they have a genetic or physical origin; or exogenous, if they arise from the social and physical environment (Figure 1) [17,26]. In general, stressors elicit behavioral, metabolic and physiological changes in animals [27]. Acute stress can occur if the stressor persists for minutes up to a few days and elicits a “fight-or-flight” response [28]. This type of stress is considered a physiological response. In contrast, long-term stressors can lead to chronic stress, which is considered a health disorder [26]. Whether acute stress becomes chronic depends on the time of exposure to the stressor and on the animal’s ability to cope with the stress [28].

Beef cattle can respond to stress through acclimatization, acclimation or temperament. In acclimatization, the animal adapts to several stressors within its natural environment, while acclimation refers to the phenotypic response of an animal facing an individual stressor [24]. Temperament refers to an animal’s reactivity to humans and the immediate environment [26].

Some cattle breeds such as Braford are more easily stressed than others, such as Angus, Brangus or Simbrah [29], highlighting the importance of temperament in animal welfare. We, therefore, recommend to extend the research on temperament of beef animals by breed. Then, the advice on the optimal beef breeds, could add to the suitability to orography and climate conditions, to the local epidemiology, also the temperament and basal behavior to human handling.

Several stressors affect cattle herds. For example, heat stress affects dairy cattle [30,31] and beef cattle [14]. Transporting animals to the slaughterhouse or a new farm can stress them [32,33]. Weaning stresses calves and is a key issue in animal welfare [34,35,36]. The present survey focuses on stressors essential for beef cattle management but rarely reviewed in the literature: management stress due to handling, social environment or weaning; nutritional stress; and thermal stress. We also review the influence of these stressors on reproductive performance and effectiveness of reproductive biotechnologies in beef herds.

### 2.2. Physiology of Stress

**Figure 2:**
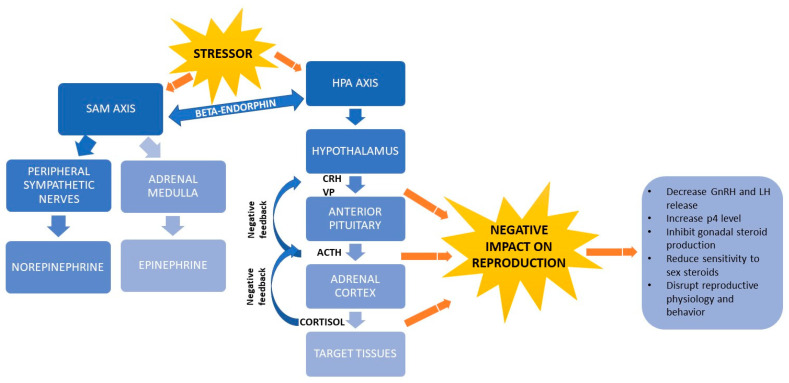
Schematic representation of stress physiology and its link with reproductive hormones. ACTH, adrenocorticotrophic hormone; CRH, corticotropin-releasing hormone; GnRH, gonadotropin-releasing hormone; HPA, hypothalamus-pituitary-adrenocortical; LH, luteinizing hormone; p4, progesterone; SAM, sympathetic-adrenal-medullary; VP: vassopresin.

Afferent pathways transmit the stress signal into various parts of the central nervous system (CNS), including the thalamus, hypothalamus and cortex. Efferent pathways are activated to give rise to a stress response [24], defined mainly by the sympathetic-adrenal-medullary (SAM) axis [18,37,38] and hypothalamus-pituitary-adrenocortical (HPA) axis [39,40] (Figure 2). Beta-endorphin release by the pituitary gland links the two axes [18].

While the SAM axis mediates acute stress responses, the HPA axis mediates both acute and chronic responses [17,41]. In the acute stress response, changes in the environment activate receptors in the body [27], which triggers the SAM axis to turn on production of two catecholamines, norepinephrine in the peripheral sympathetic nerves and epinephrine in the adrenal medulla. These hormones trigger a fight-or-flight response in which the heart rate and respiration rate increase, blood pressure rises and activity in the gastrointestinal tract decreases [37]. Epinephrine also induces glucose metabolism by activating the protein kinase AMPK and the GLUT4 transporter through an insulin increase [18]. In addition, catecholamines influence thermogenesis, lipid metabolism and insulin signaling and they regulate production of cytokines that participate in immune responses [18].

When an animal’s adaptive mechanisms can no longer cope with a stressor, the HPA axis produces metabolites [40,41] that generates energy used to trigger behavioral, autonomic, neuroendocrine and immune responses [15]. The HPA axis induces the hypothalamus to secrete corticotropin-releasing hormone (CRH) and vassopresin (VP) [40,42], which in turn induce the pituitary gland to release adrenocorticotropic hormone (ACTH). ACTH causes the adrenal cortex to secrete glucocorticoids, mainly cortisol [18]. Cortisol binds to plasma globulins, especially albumin and corticosteroid-binding globulin (CBG) and is transported by the circulatory system [40]. The strength of cortisol’s effects depends on how much and how long cortisol is secreted, how concentrated binding globulins are in the peripheral blood and receptors are in target tissue and how much glucocorticoid metabolites are broken down [43]. When the hypothalamus and anterior pituitary detect high cortisol concentrations, they exert negative feedback via VP, CRH and ACTH to inhibit further cortisol release by the adrenal cortex, thereby ending the stress response [26]. Cortisol and other glucocorticoids in cattle regulate the balance between anabolism and catabolism and under conditions of heat stress, they down-regulate the expression of lipoprotein lipase in charge of lipolysis, decrease the carbohydrate metabolism and bovine peripheral blood leukocytes amount as well as alter the expression of genes related to glycolysis and insulin-induced glucose uptake [18,24].

When a stressor activates the HPA axis and triggers secretion of CRH, ACTH, glucocorticoids, VP and opioids such as β-endorphin, the level of gonadotropin-releasing hormone (GnRH) decreases [44,45], while the levels of progesterone [46] gonadotropins, prolactin and glucagon increase [47]. In addition, glucocorticoids inhibit the pituitary production of gonadal steroids and reduce the sensitivity of target tissues to sex steroids. Arachidonic acid and its metabolites trigger the rapid initial release of LH, while protein kinase C-dependent mechanisms mediate the prolonged release of LH. Glucocorticoids reduce the release of LH by inhibiting the hydrolysis of phospholipids, thereby preventing the production of arachidonic acid. Glucocorticoids also affect the ability of gonadal steroids to regulate the pituitary production of gonadotropins [45]. In the presence of glucocorticoids, the level of gonadal steroid hormones can decline during hours or even days [48] and this decline disrupts reproductive physiology and behavior [46] and decreases feeding and appetite [20].

Glucocorticoids can influence a broad range of innate and acquired immune responses. They can induce a pro-inflammatory response [49] and acute-phase protein production [50,51]. In 2011, Cooke and Bohnert [50] first described the CRH-induced release of pro-inflammatory cytokines and acute-phase proteins in cattle. These cytokines are carried to the liver, where they trigger the synthesis of acute-phase proteins in hepatocytes, such as serum amyloid-A (SAA) and haptoglobin [52].

When a stressor persists and the HPA axis is unable to control its effects, homeostasis cannot be restored, resulting in allostatic overload [17,26]. Such overload, if chronic, harms the immune system [18], the reproductive system [15,41] and, consequently, animal welfare [26].

### 2.3. Quantification of Stress

**Figure 3:**
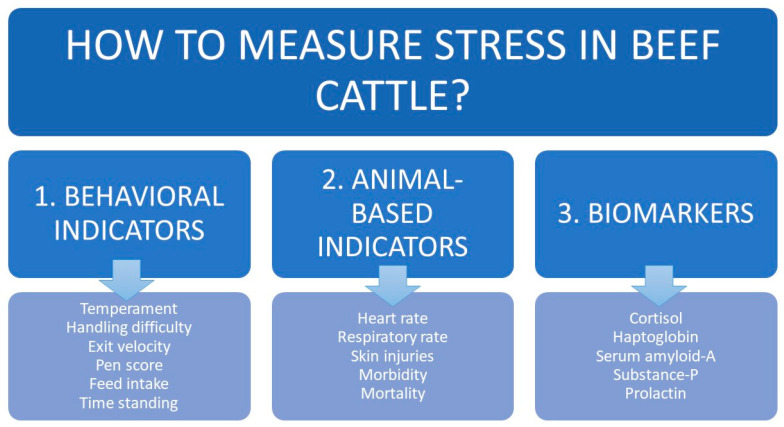
Main indicators for quantifying stress in beef cattle.

Several groups of indicators have been described to evaluate the level of stress in beef cattle (Figure 3). When used together, the indicators can provide a relatively complete picture of animal welfare.

#### 2.3.1. Behavioral Indicators

One of the first approaches to measure stress while handling beef cattle in chutes dates back to 1943 [53] and later approaches also focused on behavior [54,55,56]. In 1961, Tulloh [57] introduced the “temperament” concept and published the first “temperament score” to describe animal behavior when entering the chute, as it passed through the bail or when it was captured in the bail. Temperament scores of 1 indicate a docile animal that does not hesitate to enter the bail, while maximal scores (4 or 6, depending on the scale used) indicate an aggressive animal that is difficult to handle [57]. In 1979, Hearnshaw and colleagues developed a score for “handling difficulty” (from 0 to 5) when handling cattle in the bail: 0 is used for an animal that stands quietly and offers no resistance with only casual tail swishing, while 5 is used for “unmanageable and dangerous” animals [58]. Subsequently, scoring schemes based on animal movement have been proposed, such as by Fordyce and colleagues [59]. Grandin [60] proposed a five-point scale based on the temperament in the squeeze chute (crush). Curley and collaborators [61] combined three parameters: exit velocity, a pen score (1–5) based on behavior when the animal is penned into a small group and a chute score (1–5) based on animal behavior in the chute. Cooke et al. [19] combined a temperament score in the chute (1–5) with an exit score leaving the chute (1–5). Kasimanickam and collaborators [20] combined Grandin and Cooke’s scales to make a 0–1 scale, with 0 referring to a calm animal with slow exit and walk; and 1 referring to an excitable animal with a fast exit, jump, trot or run. Finally, an objective chute score has been described recently [62].

Additional behavioral indicators of beef cattle stress have been proposed. These include movement-measuring devices [63], a docility test [64], strain gauges [65], a race score [66], qualitative behavioral assessment [67] and a Four-Platform Standing Scale [68]. Most of these behavioral scales have been shown to be objective, repeatable and correlated with different biomarkers, including cortisol [19,61], haptoglobin [19], substance P and prolactin [20].

Other behavioral indicators have been described and are commonly used in dairy cattle but they are less useful for beef cattle, which are generally housed outside over large areas. These indicators include amount and timing of feed intake, time spent standing and time spent lying down [69].

#### 2.3.2. Animal Based Indicators

In recent years, animal-based indicators have taken priority over resource-based indicators as being more reliable and more indicative of the animal’s experience. This means that farmers often rely not only on behavioral indicators but also on physiological indicators when assessing animal welfare, such as body temperature, heart rate, respiratory rate and the presence of lesions or injuries. Stressors trigger cortisol secretion, which initiates a fight-or-flight response that increases heart rate and respiratory rate [28,37]. A feed intake index can measure stress around feeding, which may be due to chronic stress or fear of human contact or hierarchy. Analyses based on such an index have shown that hierarchical dams consume more feed and that under heat stress, animals consume more feed during the cooler parts of the day [24]. Nevertheless, feed intake indices have limited usefulness for beef cattle because the animals normally feed on pastures with scarce supplementation.

Skin injuries can indicate aggression between animals or inadequate housing conditions [70]. Morbidity and mortality rates can be used as indicators of herd welfare, although they are not always related to stressful events. Nevertheless, stressors compromise the immune system and can predispose to diseases, thereby increasing morbidity and mortality [18].

#### 2.3.3. Biomarkers

A “biomarker” is an objective indicator of a medical state that can be observed from outside the individual and can be measured accurately and reproducibly [71]. The main biomarkers of stress in cattle are cortisol, haptoglobin and serum amyloid-A. The 11-amino acid neuropeptide substance P has also been used as a biomarker [72,73]; it regulates the excitability of dorsal horn nociceptive neurons and is present in areas of the neuroaxis involved in the integration of pain and anxiety [74]. C-reactive protein (CRP) is often used as a stress biomarker in humans and dogs but only occasionally in cattle [75]. Lipopolysaccharide-binding protein, an acute-phase protein, may be less suitable as a stress biomarker because it is more closely linked to innate immunity and host defense [76].

The stress biomarkers cortisol, corticosterone and their metabolites can be measured in cattle blood, saliva, feces and hair [77,78]. They can also be assayed in urine or milk [40] but sampling these fluids in beef cattle is complex and calved dams are needed in order to obtain milk. Cortisol levels in blood or saliva reflect the animal’s recent past; levels in feces, the animal’s situation 24–48 h ago; and levels in hair, the animal’s experience weeks or months ago, depending on the rate of hair growth [77,79]. Thus, assaying cortisol in hair can be particularly useful for analyzing chronic stress. To assay cortisol in blood samples, samples must be centrifuged as soon as possible after collection and stored at −20 °C or −80 °C, where they can be kept for years [80]. Saliva samples must also be centrifuged immediately and they can be stored at room temperature for days or weeks [81] or at −20 °C or −80 °C for months or even up to a year [82]. Fecal samples must be stored at −20 °C. In feces, cortisol levels are determined by assaying its metabolite 11,17-dioxoandrostane [83] or other glucocorticoid metabolites and the results can depend on the proportions and timeframe of the metabolite’s release into feces, so any feces-based cortisol assay must first be validated against measurements in another tissue, such as blood [40,84]. Hair is easy to sample and it can be stored at room temperature indefinitely [77]. To examine the impact of interventions or experiences on animals, hair should be sampled from the baseline up to the end of the intervention or observation period. Therefore, hair must be clipped at the beginning of the study. The relationship between cortisol levels in hair and those in other tissues is unclear; levels in hair may vary with season, hierarchy or dominance [77].

Haptoglobin is a α2-globulin synthesized by the liver during the acute-phase response [85]. Healthy cattle contain negligible levels of haptoglobin but levels can increase >100-fold in the presence of stress [86]. The biomarker is assayed in blood and samples can be stored at −80 °C for months. Serum amyloid-A proteins, which are apolipoproteins associated with the high-density lipoprotein, are synthesized by the liver during the acute-phase response [52]. Although originally used as a pain biomarker [87,88,89,90], substance P can be used as a stress biomarker. Kasimanickam’s group, for example, showed that its levels were higher in aggressive dairy heifers than in mild or calm heifers [91] and that its levels increased in beef heifers after undergoing Artificial Insemination at fixed time (FTAI) [92]. Substance P can be assayed in blood using enzyme-linked immunoassays or enzyme-linked immunosorbent assays and samples can first be frozen [87,90,92]. Prolactin was originally considered a potential stress biomarker because its levels in beef heifers depend on their temperament [92] but it is not routinely used in this way because its levels are strongly influenced by dairy husbandry practices, as well as by light levels and temperature [93].

## 3. Main Stressors in the Beef Cow

**Figure 4:**
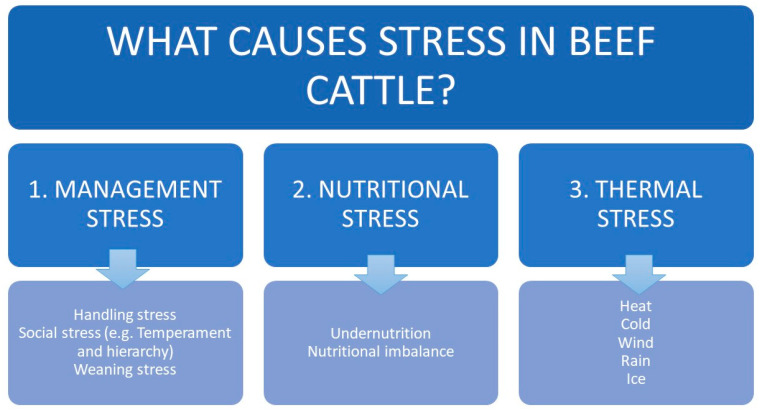
The main stressors in beef cattle herds.

Stressors in beef herds differ from those in dairy cattle production systems because the herds are housed and managed under extensive conditions with few facilities. Assuming that infectious diseases are controlled, beef herds are most likely to experience one or more of the following three types of stress (Figure 4): (1) management stress, which includes handling, social stress, herd hierarchy and weaning stress; (2) nutritional stress, which includes undernutrition and nutritional imbalance; and (3) thermal stress.

### 3.1. Management Stress

In this part we review stress that animals may experience when they are handled in the chute; when they are managed in groups, where they are exposed to social stress and hierarchies; and when they are weaned, that is, when calves are separated from their mother.

#### 3.1.1. Handling Stress

Every veterinary intervention in beef cattle, such as reproductive management, requires them to be moved into chutes. Moreover, beef cattle are less accustomed to humans than dairy cattle are, so the close contact with humans can stress them [25]. As a result, ensuring that animal handling and the facilities themselves optimize animal welfare is essential for the success of reproductive technologies.

When handled by humans and squeezed into chutes, cattle experience a fight-or-flight response, which can harm reproductive performance. In fact, when unfamiliar staff introduce animals into a chute, the animals may experience a similar level of stress as when they are branded [94]. Calm and quiet handling can reduce this stress [95]. Farms should familiarize beef cattle with chutes and facilities; they should provide for plenty of human-animal interaction (acclimation) from when animals are still calves [96]; and humans should walk quietly within the herd and cattle should pass through the chute several times a week without stress [95,96,97]. Even though temperament has a strong genetic component [59,98,99], it can be mitigated through appropriate handling and human contact.

In two experiments, Cooke et al. [19] described the impact of temperament and acclimation on stress and reproductive performance of beef cattle. In an experiment on temperament, they observed that aggressive animals showed higher plasma cortisol concentrations; reduced rates of pregnancy, calving and weaning; and reduced calf weight at birth and weaning. In the experiment on acclimation, they found that 200-day acclimation was associated with earlier onset of puberty, lower levels of haptoglobin and cortisol and slower exit velocity [19]. Other studies have reported similar results [100,101], including an association between excitable temperament in cows and lower average daily gain and carcass weight in offspring steers [100].

Facilities should be designed to reduce individual stress and the amount of handling by humans. For example, facilities where animals can be handled in groups rather than individually and where animals can be loaded into semi-circular rather than straight chutes, may induce less stress [92,95].

#### 3.1.2. Social Stress in the Beef Cow. Temperament and Hierarchy

Aggressive temperament in heifers can lead to lower pregnancy rate and delay puberty or even prevent its onset [50,102,103,104]. Conversely, quieter temperament has been shown to lead to higher rates of estrus and pregnancy, shorter time to pregnancy and lower rate of pregnancy loss [20,99,101]. Those studies also found significantly higher levels of the stress biomarkers cortisol, substance P and haptoglobin in excitable cows than in calm ones. Higher cortisol concentrations in excitable dams reduce GnRH and LH, harming their reproductive performance [102] and prolonging postpartum anestrus [44]. Moreover, excitable dams are more sensitive to environmental threats and they show signs of chronic distress, leading to lower feed intake and therefore lower body condition score (BCS). Ultimately, this can create a negative energetic balance (NEB) that that influences many physiological processes, including reproduction, negatively [20,105].

The social system of cattle is highly hierarchical [106]. This hierarchy influences feed intake, social behavior, relationships between dams and group creation [107,108]. For example, high-ranking cows in one study entered the chute before medium- and low-ranking cows during a 19-day handling period [109]. Additionally this study found that low-ranking cows had lower cortisol levels in plasma than the medium- or high-ranking cows starting from day 2 of the 19-day handling period, leading the authors to conclude that low-ranking dams may adopt a passive behavioral strategy in order to reduce their stress. Hierarchy also influences reproduction: stressed dams of low social rank produce less LH, preventing ovulation and estrus behavior [45]. However and although a dam’s social rank (i.e., hierarchy) has traditionally been thought to depend on her age and on whether she has horns, a recent study in beef cattle up to 14 years old showed that dam dominance related to its age did not significantly influence reproductive performance or success in terms of fecundity, weaning or calf weight at birth or weaning [110].

#### 3.1.3. Weaning Stress

Weaning is considered a major source of stress for beef calves, which have to face changes in the social and physical environment, the loss of the mother, the loss of the intake of milk and the loss of suckling behavior, with a superimposition of all of these stressors. Under natural conditions, the survival of the newborn depends on the establishment of a strong social bond with the dam, that once established, lasts for months. Unfortunately, little is known about the physiological and behavioral process during the diminishing of the bond to the dam [111,112]. Weaning induces behavioral (mainly increase of the frequency of vocalizations, of the general activity and of the walking frequency) and physiological reactions, which indicate detrimental effects on the welfare of these calves [113,114]. Different biochemical and hormonal markers have been found to be altered in weaned calves, such as cortisol, norepinephrine, peripheral catecholamines, acute phase proteins, the ratio neutrophils:lymphocytes and antioxidant enzyme activity of leukocytes. Calm temperament of cows influences also the behavior of calves, resulting in calm calf temperament, which may reduce weaning stress. Therefore, selection of docile cows in breeding programs may reduce calf excitement and improve calf performance [99]. Therefore, studies assessing methods to minimize weaning distress should be investigated and developed and included in practical management programs [34].

Weaning can be performed in different ways: suddenly or progressively, earlier in the calf’s life (e.g., a few weeks after birth) or later (traditionally 7 months) [34,36]. Progressive weaning, such as through the use of fences, nose-flaps or timed-contact with dams, together with restricted sucking, may best approximate the natural process in mammals [115]. Such weaning induces stress only transiently, because mother and calf quickly become accustomed to scheduled contact. Weaning stress leads to an increase in vocalizations and locomotor activity, which correlates with an increase in cortisol and weight loss [116]. Milk accumulation in the udder induces additional stress in the mother.

Although weaning is considered a major source of stress for beef calves, it is a necessary practice to ensure reproductive efficiency, accelerating rebreeding of the dam postpartum [34]. Beef cattle farms must strike a balance between weaning stress and animal welfare, because they must keep the period of postpartum anestrus short to ensure a sufficient inter-calving rate. This rate is a major economic index for the farm. Therefore, calves should not be allowed to suckle too long, since this interferes with GnRH secretion by the hypothalamus and even the mother’s seeing and smelling the calf can prevent the GnRH secretion and the LH release, prolonging anestrus [36,117,118]. Thus, weaning in beef cattle should be carried out early enough to maximize production but not too early to jeopardize animal welfare.

### 3.2. Nutritional Stress: Under Nutrition and Imbalanced Nutrition

Nutrition is important not only for calf and heifer growth but also during pregnancy: the metabolic environment during fetal development affects epigenetic modifications that influence the reproductive potential of heifers [119]. Moreover, adequate nutrition during early growth is essential for heifers to achieve puberty at an optimal moment [120].

Energy balance is the result of energy expenditure and energy intake and intake by beef cattle is sometimes reduced because of extensivity and pasture use. Undernutrition or imbalanced nutrition, leading to NEB, are therefore common stressors in beef cattle and can affect post calving estrus cyclicity [8]. NEB should always be avoided, especially pre- and post-partum and the most appropriate feed supplementation depends on altitude, latitude and orography. Energy balance can be assessed in terms of the BCS, which ranges from 1 (cachexic) to 5 (obese) [121]. A herd-level BCS of 2.5–3 appears to be generally sufficient for maintaining energy balance and supporting reproduction [8]. Lower BCS around calving is associated with longer postpartum anestrus in multiparous cows and even longer anestrus in primiparous dams. In fact, BCS at calving appears to be even more important than any change in BCS during the last trimester of pregnancy for determining the duration of postpartum anestrus [8,122,123]. The calf’s suckling can also prolong postpartum anestrus by decreasing LH release.

**Figure 5:**
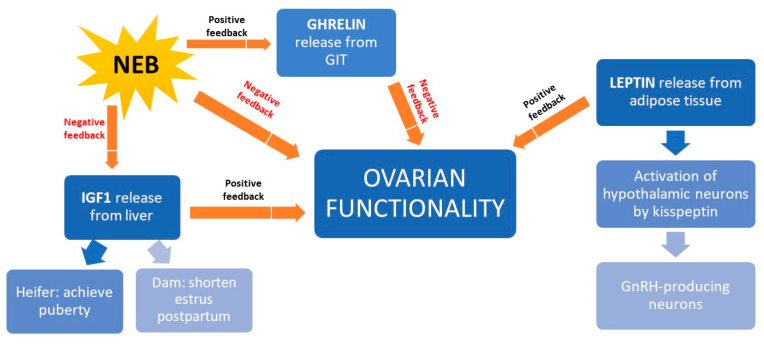
Schematic representation of how nutrition influences the production of various hormones that ultimately affect ovary function in the beef cow. GIT, gastrointestinal tract; GnRH, gonadorelin-releasing hormone; IGF1, insulin growth factor, type 1; NEB, negative energetic balance.

NEB interferes with reproduction because it inhibits the release of insulin growth factor type 1 (IGF1), thereby preventing oocyte maturation before ovulation [124] (Figure 5). In beef heifers, high IGF1 levels accelerate onset of puberty [125] and shorten postpartum anestrus [126]. Better BCS is associated with higher IGF1 levels and higher fertility rates in beef cattle [8]. In addition to IGF1, the hormone leptin also mediates the link between nutrition and reproduction. It is secreted by adipose tissue and it activates a receptor expressed on kisspeptin-expressing neurons in the hypothalamus. These neurons trigger GnRH production, so leptin is key to GnRH release [126,127]. A third hormone mediating the link between nutrition and reproduction is ghrelin, the secretion of which by the gastrointestinal tract is triggered during feed restriction or NEB [128]. Ghrelin binds to receptors in the hypothalamus neurons inhibiting GnRH release [8].

Moreover, including fatty acids in feed increases the energy density of the diet and may improve effects on reproduction in beef cattle [129]. Whether saturated or unsaturated fatty acids are more desirable in this regard is unclear [8,127,130] and further work is needed in this area. To keep postpartum anestrus short and pregnancy loss rate low, organic selenium can be administered prepartum or a diet rich in nonstructural carbohydrates or certain fatty acids can be given postpartum [131]. These nutritional strategies may contribute to uterine health, ovarian cyclicity as well as embryo survival and development.

### 3.3. Thermal Stress

Climate can be a chronic stressor of beef cattle because they usually stand outside during most of the year. Cold, heat, humidity, rain, ice and wind act as stressors affecting the endocrine system and by extension, the reproductive system [132]. Heat stress is perhaps the best studied of all climatic stressors. Heat and cold stress exert different effects, although they are interrelated through climate and the effects of each depend on seasonality, latitude and intensity [24]. Bova et al. [14] defined heat stress as a level on the temperature-humidity index (THI) that persistently lies above the thermo-neutral zone and that adversely affects the animal’s performance. THI > 72 negatively affects bulls [133], while THI > 75 negatively affects dams and has been associated with heat stress in beef cattle [134]. THI may underestimate climatic stress in beef cattle because it does not take into account exposure to radiation or wind velocity.

Heat stress can be detected based on several animal-based indicators of behavior, such as longer time spent standing, time spent looking for shaded areas, higher water consumption, changes in feeding and tendency to graze when temperatures are cooler [132]. Physiological indicators of heat stress include increased sweating, faster respiration (breaths per minute), higher panting score and higher body temperature [132]. Although heat stress affects several organs and physiological processes [135,136,137], the present review focuses on how it alters the release of reproductive hormones.

Heat stress in non-pregnant dairy cattle increases levels of progesterone in plasma [138], which in turn increases levels of cortisol, ultimately reducing LH release [139] and levels of estradiol in plasma [140]. This leads to a reduction in estrone-sulphate and increase in progestin, even in pregnant cows [141]. Heat stress also disrupts follicular waves [142], corpus luteum regression, ovulation [143,144] and oocyte quality [24]. These studies make clear that heat stress can harm reproduction in dairy cattle through numerous pathways [24].

Similar findings have been described for beef cattle. Heat stress has been associated with lower corpus luteum weight [145] and diameter [146]. It has also been associated with lower conception rate; lower concentrations of progesterone, prolactin and estradiol; and higher rate of pregnancy loss [146,147]. Higher THI has been linked to changes in estrus behavior of beef cattle, which is heavily influenced by herd hierarchy [148]. Heat stress has been linked to fewer mounts per cycle in the summer and a longer interval between mounts [149]. Cortisol levels increase more prior to calving in August than prior to calving in October and gestation is shorter in summer than in winter [150].

Heat stress increases pregnancy loss by reducing the weight and diameter of the corpus luteum, the amount of progesterone that it produces and oocyte quality. Heat stress also alters the endometrial environment, such as by up-regulating glycoprotein 2 and neurotensin, which may contribute to infertility in the summer [151]. All these changes lower the fertilization rate and reduce the quality of any resulting embryos, increasing the risk of pregnancy loss and decreasing reproductive performance. This has been observed in beef cattle [152] and dairy cattle [153,154].

In beef cattle, heat stress alters the expression of genes related to milk production, reducing the total production of milk and the protein content of the milk [155]. The resulting milk contains higher proportions of fat in general and of long, unsaturated fatty acids in particular.

## 4. Beef Cattle Stress and Reproductive Biotechnologies

The main reproductive strategy with beef cattle is natural mating. Nevertheless some farms mix natural mating with reproductive biotechnologies [156,157] Artificial insemination (AI) is increasingly applied to beef herds, after visual observation of estrus or in programs of fixed-time AI (FTAI) [156,157,158]. Under certain circumstances, embryo transfer, ovum pick-up and in vitro production of embryos (IVP) may help improve genetic merit and herd reproductive performance [159].

FTAI programs allow the insemination of many dams or heifers at once, without the need to observe estrus, which is challenging in beef herds because they are normally kept outside [157]. Several hormonal protocols have been developed to achieve high conception rates with FTAI in beef [158]. The handling required for hormonal administrations and the restraint required for insemination can induce substantial stress. Kasimanickam et al. (2014) [92] found that pregnancy rates after AI were significantly higher in calm heifers than in excitable ones. This may be because the acute stress cancels out the animal’s response to the latest GnRH administration [102], inhibiting the LH peak and ovulation. Indeed, even when ovulation occurs in more temperamental heifers, the resulting corpus luteum arises from a smaller follicle and does not produce enough progesterone to sustain the early embryo [92]. More recent work from the same researchers showed a higher rate of pregnancy per AI in calm cows (Table 1) [99]. Similarly, Cooke et al. (2017) [101] found a higher conception rate after first AI in calm beef cows than in excitable ones (Table 1). This difference in conception rate disappeared after subsequent AIs, probably due to acclimatization.

Handling stress is a particular concern for ovum pick-up or embryo transfer, which often require superovulation treatments involving several hormone administrations per day during several consecutive days [160]. Handling stress increases cortisol levels in plasma and inhibits GnRH release, interfering with the LH peak, which is critical for corpus luteum formation and sufficient progesterone production in embryo transfer [161,162]. The effectiveness of the hormonal treatments in beef cattle may be substantially increased by accustoming them to handling and by ensuring the presence of a trained veterinarian and specialized equipment during the procedures. Indeed, Biancucci et al. [163] have described a superovulation procedure for beef cattle that involves less handling and activates the HPA axis less than traditional superovulation protocols. Similarly, using a loco-regional anesthetic [164] or even sedation with xylazine or another drug [165] can reduce activation of the HPA axis during ovum pick-up, thereby mitigating stress.

Another way to improve pregnancy rates after AI in beef herds is to administer a non-steroid anti-inflammatory drug (NSAID) to act as an antiluotelytic. This can decrease pregnancy loss occurring at 10–17 days into the pregnancy [166]. The NSAID inhibits the enzymatic cascade based on cyclooxygenase (COX) enzymatic cascade, reducing the prostaglandin F2α (PGF2α) production. Some studies suggest that the effectiveness of NSAIDs depends severely on the factor “farm” [167] and that such drugs may sometimes even reduce the rate of pregnancy per AI [168], perhaps because of handling stress and temperament. In fact, temperament can modulate the effects of NSAIDs during Multiple Ovulation and Embryo Transfer (MOET) programs in beef cattle: flunixin meglumine significantly increased the pregnancy rate in temperamental dams but not in calm dams [169,170].

Temperament can also influence the success of reproductive technologies by affecting embryo viability: embryos from excitable dams in one study showed higher cortisol levels and lower viability rates than those from calm dams (Table 1) [171], while temperament improved pregnancy rate after embryo transfer in calm cows vs. excitable ones in another study [169]. This further highlights the importance of acclimation and acclimatization for reproductive procedures, specifically when implementing reproductive biotechnologies.

**Table 1:** Summary of reproductive results of calm vs. excitable beef heifers and cows summited to different reproductive biotechnological strategies.

Measured Parameter	Type of Animals	Temperament		Reference
		Calm	Excitable	
CR at FTAI	Heifers	60.30%	51.90%	Kasimanickam et al. 2014 [20]
CR at FTAI	Heifers	62.70%	53.40%	Kasimanickam et al. 2018 [99]
CR at FTAI	Cows	47.30%	41.00%	Cooke et al. 2017 [101]
Cortisol concentration	Cows	16.0 ± 2.1	12.5 ± 1.0	Macedo et al. [171]
Embryo Viability	Cows		19% less	Macedo et al. [171]
P after ET	Cows	60.20%	52.40%	Kasimanickam et al. 2019 [169]
P after ET	Cows	62.70%	49.20%	Kasimanickam et al. 2018 [170]
P after ET using NSAIDs	Cows	59.30%	56.80%	Kasimanickam et al. 2018 [170]
P after ET without NSAIDs	Cows	59.40%	46.30%	Kasimanickam et al. 2018 [170]

CR, Conception rate; FTAI, fixed-time artificial insemination; P/ET, pregnancy after embryo transfer; NSAIDs, non-steroid anti-inflammatory drug;.

Shortening the weaning time (in order to shorten postpartum anestrus) can also influence the success of reproductive biotechnologies in the cow. Weaning lasting 72 h can improve LH release and pregnancy rate during MOET programs [172]. A 96-h weaning procedure led to higher pregnancy rates in beef cattle when combined with FTAI involving GnRH, estradiol and an intravaginal device [173]. Another technique to shorten postpartum anestrus in beef cattle when performing reproductive biotechnologies is to administer estradiol. Anestrus inhibits LH release, preventing ovulation despite the presence of GnRH. Estradiol promotes LH release, improving outcomes of FTAI in beef cattle [157,173]. Nevertheless, estradiol administration is illegal in some countries. Equine Chorionic Gonadotropin (eCG) is another hormone effective for anestrus, because it promotes follicle development and growth. Especially in dams with poor BCS and postpartum anestrus, it can increase the rate of pregnancies per AI [157]. Hormonal protocols for FTAI including progesterone, GnRH and eCG have demonstrated higher pregnancy rates in several studies [172,174,175]. Similarly, a GnRH-based protocol, combined with a progesterone device, led to shorter postpartum interval until embryo transfer than natural estrus in beef dams [176].

Heat stress also influences the success of reproductive biotechnologies in beef dams: it reduces fertilization rate and embryo quality, while increasing the rate of pregnancy loss [152]. Therefore, reproductive biotechnologies are generally not performed in beef cattle during hot months. One alternative might be to use frozen embryos, as demonstrated for dairy cattle [177] or to use breeds more resistant to heat stress, such as Nellore [178].

## 5. Stress and Reproductive Efficiency of the Beef Bull

Selection of reproductive bulls is critical for success. An adequate Bull Breeding Soundness Evaluation (BBSE) should be performed before purchasing a bull or introducing it in the herd [179,180]. When several bulls live together with the same dam herd, bull hierarchy should be taken into account, because it strongly affects their sexual activity [181]: low-ranking males produce less testosterone, leading to lower sexual activity [45]. In Spain, approximately 70% of beef cattle farms have fewer than 25 cows, while only 4% have more than 100 and most farms feature only one bull per herd [182].

Of the various factors that may influence bull reproductive efficiency, space is not normally a stressor the way that it is in feedlots. Nevertheless, overcrowding may induce stress while bulls feed or mount dams [183]. More likely to induce stress in bulls in beef herds is handling, which can reduce the score on the quality of semen collected for the BBSE evaluation. Acclimation and acclimatization when performing semen extraction in reproductive centers has been demonstrated to lead to lower cortisol levels in hair. Temperament is also important: docile or calm bulls show a greater percentage of normal sperm with fewer primary defects, albeit more secondary defects, than aggressive or excitable bulls [184].

Nutrition is another stressor that affects beef bull reproductive efficiency. Undernutrition or imbalanced nutrition produce lower BCS, which is linked to poor semen quality. Excessive body fat, however, is also detrimental to bull fertility [10], so balanced nutrition is imperative to maximize welfare, semen quality and fertility. This balance should exist from an early age, since it affects age at puberty and lifetime testis size [185,186]. Moreover, diet quality also affects to Sertoli cells, seminiferous tubes and other testis structures involved in semen production [10]. During the mating season, leptin, insulin, growth hormone and IGF1 signal to the hypothalamus that environmental conditions are adverse and this reduces the secretion of gonadrotopic hormones, leading in turn to a reduction of germ cell proliferation in the testes [10]. As a result, bulls lose body weight and their scrotal circumference (SC) shrinks by approximately 2 cm, which correlates with lower sperm concentration in semen and lower semen quality [187]. Thus, adequate nutrition during bull growth and reproduction is of great importance.

Heat stress also affects bull reproductive efficiency by reducing sperm quality [188,189]; heat promotes the formation of reactive oxygen species that damage sperm DNA and heat can inhibit the production of antioxidants that usually protect sperm from oxidative attack [190]. THI > 60 may be associated with reduced semen volume, number of semen doses per ejaculate and sperm concentration, although whether this translates to lower fertilization rate and blastocysts viability is unclear [191]; THI 50–60 appears to be optimal for semen production [192]. Heat stress can be mitigated by avoiding reproductive strategies during times of higher THI; by installing cooling systems, shades, water vaporization and other tools of dairy husbandry; and by choosing bull breeds adapted to the climate area [190]. Season can affect bull reproductive efficiency, even though the cow is considered a “low seasonal” species: seasonal variation in sperm concentration was observed in an analysis of more than 70,000 ejaculates collected during 31 years [188]. Sperm output was highest during the summer months, which may have been due to the longer day length in spring; testosterone concentrations were highest in spring and summer and this variable is associated with semen quality [10]. Indeed, one study found that semen collected in the spring led to a higher proportion of advanced blastocysts after in vitro fertilization than semen collected in the winter or summer [133].

Even if bulls are normally handled much less than dairy cows, they may experience substantial stress during semen collection. An artificial vagina is normally used in commercial bull reproduction centers but electroejaculation is usually used in the field [193]. Semen volume and sperm viability are usually better after collection using an artificial vagina [194] but this technique requires a long training period, not required for the electroejaculation [195]. Electroejaculation produces an acute stress response: it increases cortisol levels in plasma and the number of vocalizations in the chute [196,197]. To what extent the procedure could be painful, however, is unclear, since it does not appear to increase levels of substance P [196] and any observed discomfort could be attributable to the technician management [193]. Caudal epidural anesthesia may mitigate any pain associated with electroejaculation [197] but this must be verified in further studies. However, electroejaculation is illegal in some countries if conducted in the absence of sedation or analgesia [198,199]. For this reason, less stressful techniques have been proposed for semen collection, such as transrectal massage [193]. This technique seems to be suitable for 80% of bulls and this percentage rises if the procedure includes acclimation but the sperm motility and viability are significantly lower than those in samples collected by electroejaculation.

Any stress that affects sperm quality can have long-term effects on bull reproductive efficiency, given that spermatogenesis takes about 60 days [200,201]. This is particularly important to remember if the primary reproductive strategy is natural mating or if the herd contains only one bull.

## 6. Conclusions

Once infectious diseases are controlled and optimum feeding programs implemented, we need to be aware of the fact, that stress situations have impact on secretion of gonadotropins and consequently reproductive efficiency of beef cattle and bulls. Therefore, stressors which are frequently routine situations in these herds (passing through chutes, weaning, climate, social regrouping...) and specific control strategies, need to be foreseen, trying to minimize their impact. Fertility of breeding bulls are the key factor in herds which include bull mating. Hence, besides general management, stress management needs also to be considered when handling bulls. Excitable adults have decreased reproductive efficiency and this excitability is strongly linked to individual temperament. This temperament can be detected early in life and could be used as a tool in selection process for both, cows and sires. Thus, opportunities exist, to improve sustainability of beef farms, through stress management strategies and through selection. Finally, when implementing reproductive biotechnologies, controlling specifically the stress with acclimation strategies is associated with improved results.
